# Primidone blocks RIPK1-driven cell death and inflammation

**DOI:** 10.1038/s41418-020-00690-y

**Published:** 2020-12-03

**Authors:** Theresa Riebeling, Kunzah Jamal, Rebecca Wilson, Benedikt Kolbrink, Friedrich Alexander von Samson-Himmelstjerna, Caroline Moerke, Laura Ramos Garcia, Eileen Dahlke, Friederike Michels, Fred Lühder, Domagoj Schunk, Philipp Doldi, Bartosz Tyczynski, Andreas Kribben, Charlotte Flüh, Franziska Theilig, Ulrich Kunzendorf, Pascal Meier, Stefan Krautwald

**Affiliations:** 1 Department of Nephrology and Hypertension, University Hospital Schleswig-Holstein, 24105 Kiel, Germany; 2 The Breast Cancer Now Toby Robins Research Centre, The Institute of Cancer Research, Fulham Road, London, SW3 6JB UK; 3 Institute of Anatomy, Christian-Albrechts-University Kiel, 24118 Kiel, Germany; 4 Department of Neurosurgery, University Hospital Schleswig-Holstein, 24105 Kiel, Germany; 5 Institute for Neuroimmunology and Multiple Sclerosis Research, University Medical Center Göttingen, 37075 Göttingen, Germany; 6 Department of Emergency Medicine, University Hospital Schleswig-Holstein, 24105 Kiel, Germany; 7 Medizinische Klinik und Poliklinik I, Ludwig-Maximilians-University Munich, 81377 Munich, Germany; 8 Department of Nephrology, University Hospital Essen, University of Duisburg-Essen, 45147 Essen, Germany; 9 Present Address: DDR Biology, Bioscience, Oncology R&D, AstraZeneca, Cambridge, UK

**Keywords:** Cell death and immune response, Translational research

## Abstract

The receptor-interacting serine/threonine protein kinase 1 (RIPK1) is a key mediator of regulated cell death and inflammation. Recent studies suggest that RIPK1 inhibition would fundamentally improve the therapy of RIPK1-dependent organ damage in stroke, myocardial infarction, kidney failure, and systemic inflammatory response syndrome. Additionally, it could ameliorate or prevent multi-organ failure induced by cytokine release in the context of hyperinflammation, as seen in COVID-19 patients. Therefore, we searched for a RIPK1 inhibitor and present the aromatic antiepileptic and FDA-approved drug primidone (Liskantin®) as a potent inhibitor of RIPK1 activation in vitro and in a murine model of TNFα-induced shock, which mimics the hyperinflammatory state of cytokine release syndrome. Furthermore, we detected for the first time RIPK1 activation in the respiratory tract epithelium of hospitalized patients who tested positive for SARS-CoV-2 infection. Our data provide a strong rationale for evaluating the drug primidone in conditions of hyperinflammation in humans.

## Introduction

The scaffolding function of the receptor-interacting serine/threonine protein kinase 1 (RIPK1) regulates pro-survival signaling and inflammatory gene expression, while its kinase activity paradoxically mediates caspase-8-dependent apoptosis and RIPK3-mixed-lineage kinase domain-like pseudokinase (MLKL)-dependent necroptosis [1]. The transition between the scaffolding and kinase functions of RIPK1 is regulated by (de)ubiquitylation and (de)phosphorylation processes [2]. RIPK1 has been implicated downstream of various immune receptors [3]; however, most of our understanding regarding RIPK1 regulation originates from studies on tumor necrosis factor (TNF) signaling. In response to TNFα, ligand-induced TNF receptor 1 (TNFR1) trimerization leads to the assembly of a large receptor-bound signaling complex, termed complex I [4]. This complex comprises several factors, including TRADD, TRAF2/5, RIPK1, and E3 ubiquitin ligases such as cIAP1/2 and the LUBAC complex, which are sequentially recruited [5]. The LUBAC complex performs linear ubiquitination of RIPK1 and TNFR1, which allows for the subsequent interaction with the trimeric IκB kinase complex (IKK) via the polyubiquitin-binding adapter subunit IKKγ/NEMO [6]. Full activation of complex I requires the phosphorylation of IKK2 by TAK1, a ubiquitin-dependent kinase that associates with K63 polyubiquitin chains via TAB2 and TAB3 [7]. These components consequently activate pro-inflammatory and pro-survival signaling via NF-κB- and MAPK-signaling (p38 and JNK) [8].

RIPK1 holds a key position in the transition from TNFR1-mediated pro-survival to cell death signaling; therefore its regulation has been of particular interest in recent studies. Besides the well-described ubiquitination events, it has also been reported that RIPK1 activation is inhibited through direct phosphorylation by IKKα/β and MK2 [9, 10]. These kinases affect RIPK1 binding and dissociating from the death-inducing complex I components, restricting the formation of a secondary cytosolic death-inducing complex IIb containing RIPK1, FADD, and caspase-8 [11]. Similarly to the death-inducing complex IIa that mediates RIPK1-independent apoptosis, complex IIb can induce RIPK1-dependent apoptosis (RDA) [12]. This form of cell death can shift towards RIPK3-MLKL-mediated necroptosis in caspase-8-deficient or inhibited conditions [6]. Even though to date no substrate has been described for RIPK1, with the exception of RIPK1 itself, the development of antibodies detecting RIPK1 autophosphorylation at S166 has contributed meaningfully to monitoring the kinase active state of RIPK1 [13, 14].

In experimental models of human disorders, a wide range of infectious, autoimmune, and inflammatory diseases including the systemic inflammatory response syndrome (SIRS), are driven by RIPK1 kinase activity (reviewed and summarized in [15]). Furthermore, RIPK1-dependent necroinflammation, independent of necroptosis, has been implicated in various human pathologies, including hepatic and renal diseases as well as sepsis [16]. This state of affairs makes RIPK1 a promising target for therapeutic interventions [17]. Unfortunately, the highly efficient RIPK1 inhibitor, 5-(indol-3-ylmethyl)-3-methyl-2-thio-hydantoin, and the more stable variant 5-[(7-chloro-1H-indol-3-yl)methyl]-3-methylimidazolidine-2,4-dione, compounds termed necrostatin-1 (Nec-1) and 7-Cl-O-Nec-1 (Nec-1_s_), respectively, are limited by their very short half-life in vivo [18–20]. These compounds have failed to gain clinical relevance due to further drawbacks such as poor metabolic stability, and have proven unsuitable for application in humans [18]. Furthermore, the specificity of Nec-1 has been questioned, as it also inhibits other types of regulated cell death, such as ferroptosis [21]. A few small-molecule inhibitors of RIPK1 that offer high selectivity have been developed by different companies and have successfully entered phase I/II clinical trials in humans (source: ClinicalTrials.gov), but so far no pharmacological inhibitor of RIPK1-mediated cell death is in clinical use [22]. Nevertheless, necrostatins have been widely used to define the role of necroptosis and RIPK1 in suitable animal models of human diseases. Such studies have examined the role of the kinase in sepsis, inflammatory bowel disease (IBD), and renal ischemia-reperfusion injury (IRI) [23–25]. IRI is inevitable after kidney transplantation and also contributes to acute kidney injury, myocardial infarction, and stroke [26–28]. Humans with RIPK1 deficiency develop recurrent infections, IBD, and progressive polyarthritis, indicating that RIPK1 functions are essential for maintaining tissue homeostasis [29]. Additionally, pharmacological inhibition of RIPK1 kinase activity in multiple preclinical animal models has demonstrated the eminent impact of RIPK1 kinase-dependent cell death modalities in triggering inflammatory injuries [30, 31], making RIPK1 a promising therapeutic target.

Previously, we screened a collection of FDA-approved drugs to assess their ability to prevent RIPK1-mediated cell death and inflammation [32]. Interestingly, in the course of our research, we came across an aromatic antiepileptic drug, primidone (Liskantin®), which fulfilled our criteria. In contrast to the anticonvulsant phenytoin (Phenhydan®), which inhibits ongoing cell death processes by affecting cell membrane lipid raft formation, primidone represents a potent inhibitor of RIPK1. Primidone prevents RDA, necroptosis, and hyperinflammation effectively. We confirmed that our screening strategy leads to functionally valuable in vivo results by testing primidone for its ability to protect against RIPK1-driven inflammation in a murine model of SIRS. Furthermore, our immunohistochemical analyses of pharyngeal epithelial cell samples from COVID-19 patients suggest that RIPK1 activation, and by implication, RIPK1-induced inflammatory cell death (ICD) may contribute to the course of SARS-CoV-2-induced infection. Our findings might offer a novel therapeutic strategy for managing this pandemic. In this context, an application to conduct a clinical trial is currently being submitted to the EudraCT (European Union Drug Regulatory Authorities Clinical Trials Database).

## Materials and methods

### Cell lines and reagents

L929, NIH3T3, HT-29, U937, HT-1080, HEK293T, and Jurkat cells were obtained from American Type Culture Collection (Manassas, VA, USA). CRISPR/Cas9-edited *Ripk1*-deficient L929 and *RIPK1*-deficient HT-1080 cells were generated as described previously [26]. The used guide RNAs were 5′-TCCCGAAGCCTCCGCTGTCTAGG-3′ for murine *Ripk1* and 5′-CGGCTTTCAGCACGTGCATCAGG-3′ for human *RIPK1*, respectively. Mouse dermal fibroblasts (MDF) that were *Mlkl*-deficient and reconstituted with the phosphomimetic *Mlkl* S345D mutant (*Mlkl*^S345D^) under a doxycycline-inducible promoter were a kind gift from James M. Murphy and have been described before [33]. The MDF, L929, NIH3T3, HEK293T, and HT-29 cell lines were cultured in Dulbecco’s modified Eagle’s medium (DMEM, Gibco®; Thermo Fisher Scientific, Schwerte, Germany) supplemented with 10% (vol/vol) fetal calf serum (FCS, PAN-Biotech GmbH, Aidenbach, Germany), 100 U/ml penicillin, and 100 μg/ml streptomycin (Merck Millipore GmbH, Darmstadt, Germany). For HT-1080 cells, the medium was additionally supplemented with MEM NEAA (Gibco®; Thermo Fisher Scientific). The Jurkat cells were cultured in RPMI 1640 medium (Gibco®; Thermo Fisher Scientific) supplemented with 5% (vol/vol) FCS, 100 U/ml penicillin, and 100 μg/ml streptomycin. The U937 cells were cultured in RPMI 1640 medium supplemented with 10% (vol/vol) FCS, 100 U/ml penicillin, 100 μg/ml streptomycin, 1 mM sodium pyruvate and 0.25% D-(+)-glucose (Sigma-Aldrich Chemie GmbH, Taufkirchen, Germany).

Mouse embryonic fibroblasts (MEFs) were generated in our lab from E13.5 embryos. After the placenta, yolk sac, head, and dark red organs had been removed, the embryos were finely minced and digested for 20 min in 0.25% trypsin. A single-cell suspension was seeded for culture. Primary MEFs were cultured in DMEM supplemented with 10% FCS, 50 U/ml penicillin, 50 µg/ml streptomycin, and 7 µl/l β-mercaptoethanol. All cell lines were cultured in a humidified 5% CO_2_ atmosphere at 37 °C. Negativity for mycoplasma was routinely checked using the MycoAlert^™^ Mycoplasma Detection Kit (Lonza, Cologne, Germany).

Recombinant purified TNFα (570104) was purchased from BioLegend (Amsterdam, the Netherlands). Fas ligand (FasL, ALX-522-020) and FLAG-tagged TNFα (ALX-522-008) were obtained from Enzo Life Sciences GmbH (Lörrach, Germany), the bivalent SMAC mimetic SM-164 (A15800) was purchased from AdooQ Biosciences (via Hölzel Diagnostika, Cologne, Germany), the pan-caspase inhibitor zVAD-fmk (4026865) was purchased from Bachem (Weil am Rhein, Germany), Nec-1_s_ (2263-1-BV) was obtained from BioCat (Heidelberg, Germany); cycloheximide (C4859), phenytoin (D4505), and primidone (P7295) were purchased from Sigma-Aldrich. Liskantin® and Phenhydan® were obtained from Desitin Arzneimittel GmbH (Hamburg, Germany), necrosulfonamide (NSA) (480073), and GSK’872 were obtained from Calbiochem® (530389, Merck Millipore GmbH).

### Mice

The mice used in the IRI model were 8-week-old C57BL/6JRj male mice. For the SIRS model, 8-week-old BALB/cAnNRj female mice were used. The animals were purchased from Janvier Labs (Saint Berthevin, France), and housed in the Central Animal Facility of the University Hospital Schleswig-Holstein (Kiel, Germany), and received standard chow and water ad libitum and were held at a 12-h day–night cycle. All in vivo experiments were conducted in accordance with the animal protection regulations of the local authorities and have been approved by the Ministry of Energy, Agriculture, the Environment, Nature and Digitalization of Schleswig-Holstein, Germany [AZ: V242-29444/2019 (59-5/19)].

### Flow cytometry analysis of cell death

Phosphatidylserine exposure to the outer cell membrane of apoptotic cells or inner plasma membrane of necrotic cells and the incorporation of 7-amino-actinomycin D (7-AAD) into necrotic cells was quantified using flow cytometry. After the cells had been stimulated under the indicated conditions, single-cell suspensions were stained using the APC Annexin V Apoptosis Detection Kit with 7-AAD according to the manufacturer’s instructions (BioLegend, 640930). The samples were analyzed using an FC-500 flow cytometer (Beckman Coulter GmbH, Krefeld, Germany).

### Western blot assay

For immunoblotting, 1 × 10^5^ adherent or 5 × 10^5^ suspension cells were seeded in 6-well plates and 24 h later treated as indicated. Thereafter, the cells were harvested, washed, and lysed in ice-cold modified Frackelton buffer [10 mM Tris-HCl (pH 7.5), 50 mM NaCl, 1% Triton X-100, 30 mM Na_4_P_2_O_7_, 50 mM NaF, 100 μM Na_3_VO_4_, 2 μM ZnCl_2_, and 1 mM C_7_H_7_FO_2_S (PMSF)]. Insoluble material was removed by centrifugation (14,000 × *g*, 10 min, 4 °C), and protein concentrations were quantified using a commercial Bradford assay kit (Bio-Rad GmbH, Munich, Germany) according to the manufacturer’s instructions. Equal amounts of protein (20 μg per lane) were resolved by reducing SDS-PAGE and transferred to a polyvinylidene fluoride membrane (GE Healthcare Life Sciences; Freiburg, Germany). The membranes were probed with specific primary antibodies and corresponding secondary horseradish peroxidase-linked polyclonal antibodies obtained from OriGene Technologies (R1348HRP and R1454HRP, Herford, Germany), Promega (V8051, Mannheim, Germany) and dianova (112-035-003, Hamburg, Germany). Immune complexes were detected by enhanced chemiluminescence. Autoradiographs were generated using Amersham Hyperfilm MP high-performance autoradiography films (GE Healthcare 28906842) and developed with a Curix 60 X-ray film processor (AGFA, Mortsel, Belgium). To re-probe the same membrane, the membrane was stripped using a commercial stripping buffer (Thermo Fisher Scientific) before incubation with a different primary antibody.

### Antibodies

The following primary antibodies were used for immunoblotting at 1:1000 dilution unless specified otherwise: anti-RIPK1 (3493), anti-phospho-RIPK1 (human) (65746), anti-phospho-RIPK1 (murine) (31122), anti-RIPK3 (human) (13526), anti-phospho-RIPK3 (human) (93654), anti-phospho-RIPK3 (murine) (57220), anti-IκBα (9242), anti-phospho-IκBα (2859), anti-NF-κB (6956), anti-phospho-NF-κB (3033), anti-MK2 (3042), anti-phospho-MK2 (3007), anti-p38 (9212), anti-phospho-p38 (9215), anti-JNK (9252), anti-phospho-JNK (4668), anti-caspase-8 (9746), anti-CYLD (8462) all from Cell Signaling Technology (Frankfurt, Germany), anti-MLKL (MABC604) from Merck Millipore GmbH, anti-SHARPIN (14626-1-AP) from Proteintech (Manchester, UK), anti-TRADD (610572) from BD Biosciences (Wokingham, UK), anti-human phospho-MLKL (ab187091), anti-murine phospho-MLKL (ab196436), anti-TNFR1 (ab205718) all from Abcam (Berlin, Germany), anti-HSP90 (ADI-SPA-835, dilution 1:3000) from Enzo Life Sciences, anti-RIPK3 (murine) (2283, dilution 1:3000) from ProSci (via Hölzel Diagnostika), anti-FADD (sc-6036), anti-caspase-8 (sc-6136) both from Santa Cruz Biotechnology (Heidelberg, Germany), anti-β-Actin (A5441), anti-FLAG (F3165) both from Sigma-Aldrich and anti-HA (11867423001) from Roche Pharma AG (Reinach, Switzerland).

### Immunoprecipitation assay

HEK293T cells were transiently transfected with the indicated constructs using GeneJuice® transfection reagent (Merck Millipore GmbH) according to the manufacturer’s instructions. All constructs used for transient transfection experiments had been cloned into the pcDNA3 mammalian expression vector (Invitrogen, Thermo Fisher Scientific) and sequence-verified. The HA-RIPK1 and FLAG-RIPK1 construct encoded full-length human RIPK1; the FLAG-RIPK1 ΔRHIMspacerΔDD construct (aa 1-510 + 553-581) encoded a RIPK1 mutant deficient for the oligomerization motifs RIP homotypic interaction motif (RHIM) domain and death domain (DD) [34].

For immunoprecipitation (IP), 1 × 10^6^ suspension (U937) or 5 × 10^5^ adherent cells (HT-1080, NIH3T3, HEK293T) were treated with the indicated substances and harvested at the end of the respective incubation times, then washed and lysed using IP buffer (50 mM Tris-HCl (pH 7.4), 150 mM NaCl, 1% NP-40, 0.25%, Na-deoxycholate, 1% Triton X-100, 1 mM EDTA, 100 μM Na_3_VO_4_, 1 mM PMSF) on ice for 20 min. The cell lysates were clarified at 4 °C for 10 min at 14,000 × *g* and incubated over-night with the following antibodies: anti-HA (11867423001, Roche Pharma AG), anti-FLAG (F3165, Sigma-Aldrich), anti-FADD (sc-6036, Santa Cruz Biotechnology), or anti-caspase-8 (9746, Cell Signaling Technology). Pulldown was performed with μMACS™ Protein G MicroBeads and μ Columns (Miltenyi Biotec GmbH, Bergisch Gladbach, Germany) according to the manufacturer’s instructions. Elution was performed with 40 μl 95 °C SDS loading buffer. Complete sample volume was loaded on an SDS-PAGE for immunoblotting analysis. The blots were visualized using VeriBlot detection reagent (ab131366, Abcam).

### Kinase binding assay

Binding of small-molecule inhibitors to RIPK1 was performed following a protocol based on Drug Affinity Responsive Target Stability (DARTS) methodology [35]. Briefly, U937 cells were lysed in IP buffer (w/o PMSF). The protein concentration of the clarified lysate was adjusted to 2.5 mg/ml, and the lysate was divided and pre-incubated with 1 mM primidone, 20 µM Nec-1_s_, 2.5 µM GSK’872, 5 µM NSA, or vehicle at room temperature for 20 min. Digestion was performed using 3.3 µg/ml thermolysin (T7902, Sigma-Aldrich) for 5 min. The reaction was stopped by adding SDS loading buffer and boiling the samples at 95 °C for 5 min. The digestion fragments were analyzed using western blotting.

### Kinase activity assay

Recombinant human active RIPK1 (aa 1-327) (R07-11G, SignalChem., Richmond, Canada) was pre-incubated with 1 mM primidone, 20 µM Nec-1_s_, 2.5 µM GSK’872 or vehicle alone in Kinase Dilution Buffer IV [5 mM MOPS (pH 7.2), 2.5 mM β-glycerol-phosphate, 4 mM MgCl_2_, 2.5 mM MnCl_2_, 1 mM EGTA, 0.4 mM EDTA, 50 µM DTT, 50 ng/ml BSA, SignalChem] for 15 min. ATP was added to a final concentration of 50 µM, and the kinase reaction was allowed to proceed for 4 h at room temperature. A total of 2 µg kinase was used for each 20 µl reaction. RIPK1 kinase activity was assessed using the ADP-Glo^™^ Kinase Assay kit (Promega) according to the manufacturer’s instructions. Luminescence was measured using a Mithras LB 940 microplate reader (Berthold Technologies, Bad Wildbad, Germany).

### Kidney IRI

The mice were provided with a drinking solution containing either 2.875 mM primidone or an equivalent amount of dimethyl sulfoxide (DMSO) as a vehicle control in their regular drinking water for 5 days prior to IR surgery until the end of the reperfusion phase. Murine kidney IRI was performed via a midline abdominal incision and bilateral renal pedicle clamping for 37 min using microaneurysm clamps (Aesculap Inc., Center Valley, PA, USA). Throughout the surgical procedure, the mice were kept under isoflurane narcosis, and their body temperature was maintained at 36–37 °C by continuous monitoring using a temperature-controlled, self-regulated heating system (Fine Science Tools, Heidelberg, Germany). After the clamps had been removed, kidney reperfusion was confirmed visually before the abdomen was closed in two layers using standard 6-0 sutures. After 48 h reperfusion, the mice were sacrificed, blood samples were obtained by retrobulbar puncture and the organs were collected for analysis.

### SIRS model

Each animal received a single bolus of 1 mg murine TNFα (575208, BioLegend) per kg body weight dissolved in a volume of 200 µl PBS by tail vein injection. 15 min before TNFα application, the mice were given a single intraperitoneal (i.p.) injection (total volume per mouse was 200 μl) of either 2.5% DMSO in PBS (vehicle) or 6.25 mg primidone/kg body weight (as indicated). Body temperature was monitored using a rectal probe (BIO-TK8851 thermometer with BIO-BRET3, BiosebLab., Vitrolles, France).

### Histology

Freshly obtained kidney and lung samples were fixed in 4.5% neutral-buffered formaldehyde and embedded in paraffin. The sections were dewaxed, rehydrated, and subjected to Masson trichrome staining (kidney) or hematoxylin and eosin staining (lung) according to routine protocols. The sections were dehydrated and mounted using DePeX mounting medium (Serva, via Merck Millipore GmbH). Staining was evaluated in a blinded manner using a Leica Axiovert microscope and Axio Vision SE64 Rel 4.9 software (Leica Microsystems, Wetzlar, Germany). Mild sharpening, contrast enhancement, and gamma adjustment were performed for the data presentation.

### TUNEL fluorescence assay

To analyze cell death in the tissue sections, a TdT-mediated dUTP nick end labeling (TUNEL) assay was performed using a fluorescence-based detection kit according to the manufacturer’s instructions (G3250, Promega). Briefly, tissue sections were dewaxed, rehydrated, fixed in 4% paraformaldehyde, and permeabilized with Proteinase K for 10 min at room temperature. Following this, the sections were equilibrated with the provided buffer for 10 min and labeled with the TdT reaction mix for 60 min at 37 °C in a humidified dark environment. To stop the labeling reaction, the sections were incubated with the provided stopping buffer for 15 min at room temperature in the dark. The sections were then washed with PBS for 5 min, and mounted with Shandon^™^ Immu-Mount^™^ (Thermo Fisher Scientific). Fluorescence micrographs were acquired with a ×20 and ×40 objective magnification using a standard fluorescein filter set to view the green fluorescence at 520 nm wavelength with a Leica Axiovert microscope and Axio Vision SE64 Rel 4.9 software. Mild sharpening, contrast enhancement, and gamma adjustment were performed for data presentation. TUNEL-positive cells were quantified manually by two blinded observers by evaluating eight randomly selected fields of view per slide.

### Plasma parameters

Using a heparinized capillary tube, whole blood was collected from the retrobulbar capillary bed. Plasma was obtained by centrifugation. Creatinine, urea, and lactate dehydrogenase (LDH) concentrations were measured photometrically at the Central Laboratory of the University Hospital Schleswig-Holstein.

### Clinical samples

Patients who had tested positive for SARS-CoV-2 within the last 48 h prior to sample acquisition and who had been hospitalized for displaying typical prominent clinical symptoms (fever, shortness of breath) were included. Negative controls were obtained from healthy individuals who had tested negative for SARS-CoV-2 infection. Ethical approval for this study was obtained from the local ethics committee (Medical Faculty, Christian-Albrechts-University of Kiel, Germany, AZ: D 495/20). All patients and controls participating in the study were informed of their rights as well as the risks and benefits of sample and data collection, and gave informed written consent.

### Sample preparation and immunocytochemistry

Cell smears taken from the oropharyngeal epithelium were fixed in 4.5% formalin, blocked with horse serum, permeabilized with Triton X-100 and stained for phospho-RIPK1 using an anti-phospho-RIPK1 antibody (44590, Cell Signaling Technology) and Alexa Fluor® 488-AffiniPure Donkey Anti-Rabbit IgG (711-545-152, Jackson ImmunoResearch Laboratories, West Grove, PA, USA). Slides were mounted using ImmunoSelect® Antifading Mounting Medium with DAPI (SCR-038448, dianova). Imaging was performed using a Zeiss Axio Imager Z1 fluorescence microscope and AxioVision Rel. 4.8 software (Carl Zeiss GmbH, Jena, Germany). Figures were prepared using Fiji/ImageJ software [36]. Grayscale images were assigned the respective pseudocolor, and channels were merged. Magnification insets (×2) were produced using the ImageJ macro “Zoom_in_Images_and_Stacks”. Gamma correction (gamma-value = 0.9) was uniformly applied to display images for publication.

### Case histories of tested individuals

These data are presented as Supplementary Information.

### Statistical data analysis and image presentation

For all experiments, differences between datasets were considered statistically significant when *p* values were <0.05. Statistical comparisons were performed using Student’s *t* test unless otherwise specified in the figure legend. Asterisks are used in the figures to specify the statistical significance (*p < 0.05; **p < 0.01; ***p < 0.001). The results are presented as means ± standard deviations (SD) unless indicated otherwise. Graphs were generated using GraphPad Prism software; figures were arranged using CorelDRAW software.

## Results

### Primidone inhibits regulated cell death

**Fig. 1:**
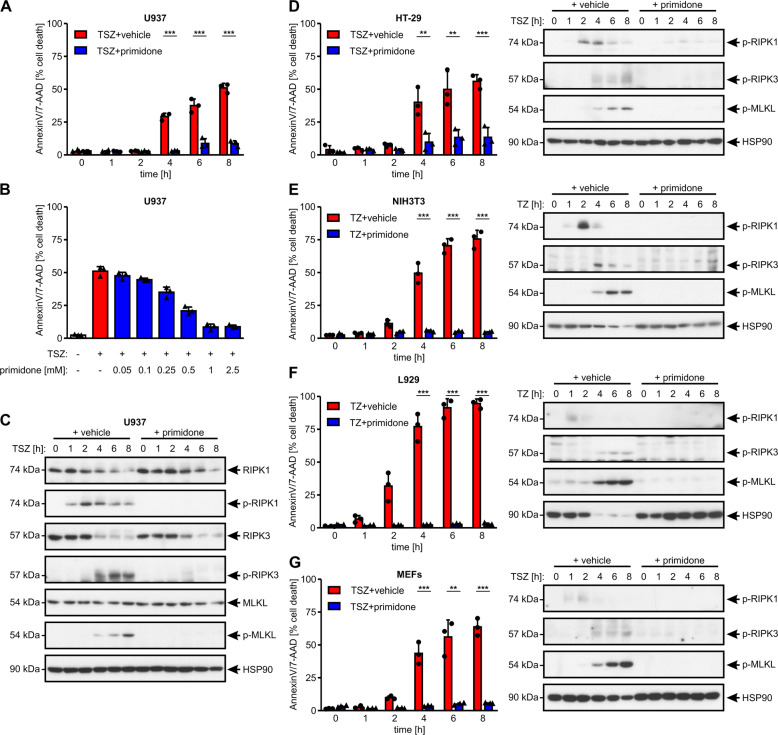
The inhibitory properties of primidone in the course of RIPK1-mediated necroptosis are species-independent. **A** Human U937 cells were stimulated at 37 °C for up to 8 h with 100 ng/ml TNFα + 1 μM SMAC mimetic SM-164 + 25 μM zVAD (TSZ) in the absence or presence of 1 mM primidone, wherein the drug was added 30 min before the induction of necroptosis. **B** U937 cells were treated for 8 h with 100 ng/ml TNFα + 1 μM SMAC mimetic SM-164 + 25 μM zVAD (TSZ) in the absence or presence of primidone (concentrations indicated), wherein the drug was added 30 min before the induction of necroptosis. Necroptotic cell death was quantified by FACS analysis using 7-AAD and phosphatidylserine accessibility (Annexin V staining) as markers. Each graph shows the mean ± SD; *n* = 3 independent repeats. Each sample analyzed in the time course of this experiment was split for the corresponding western blotting [illustrated in (**C**)]. **C** The expression levels and activation status (phosphorylation) of the indicated necrosome member RIPK1, RIPK3, and MLKL after the induction of necroptosis in the absence or presence of primidone were analyzed by western blotting, using the indicated specific antibodies. The blots were stripped and re-probed with an antibody against HSP90 as the loading control. For each cell line illustrated herein, one representative cropped blot of three independent experiments is shown. Human HT-29 cells (**D**) and primary MEFs (**G**) were treated as described under (**A**) and the activation status of the indicated necrosome members was analyzed as described under (**C**). Murine NIH3T3 (**E**) and L929 cells (**F**) were stimulated at 37 °C for up to 8 h with 100 ng/ml TNFα + 25 μM zVAD (TZ) in the absence or presence of 1 mM primidone, wherein primidone was added 30 min before the induction of necroptosis. Cell death was quantified and detection of the activation status (phosphorylation) of the indicated necrosome members was detected as described before.

Initially, we screened a collection of pharmaceuticals to assess their ability to modulate RIPK1-mediated cell death, and focused in particular on aromatic antiepileptic drugs such as carbamazepine, phenobarbital, lamotrigine, and primidone. By using the U937 pro-monocytic human myeloid cells we found that primidone (Fig. S1A shows its chemical structure) protected this cell line from RIPK1-mediated necroptosis (Fig. 1A) in a dose-dependent manner (Fig. 1B). Mechanistically, primidone treatment suppressed activation and phosphorylation of RIPK1 (p-S166), RIPK3 (p-S227), and MLKL (p-S358) in U937 cells (Fig. 1C). Notably, primidone not only inhibited TNFα-induced activation of the necrosome members RIPK1, RIPK3, and MLKL in U937 cells, but also in human HT-29 cells (Fig. 1D), and murine NIH3T3 (Fig. 1E) and L929 cells (Fig. 1F). The inhibitory properties of the drug on TNFα-mediated cell death were also evident in primary MEFs (Fig. 1G), expanding this observation to multiple cell types and species.

**Fig. 2:**
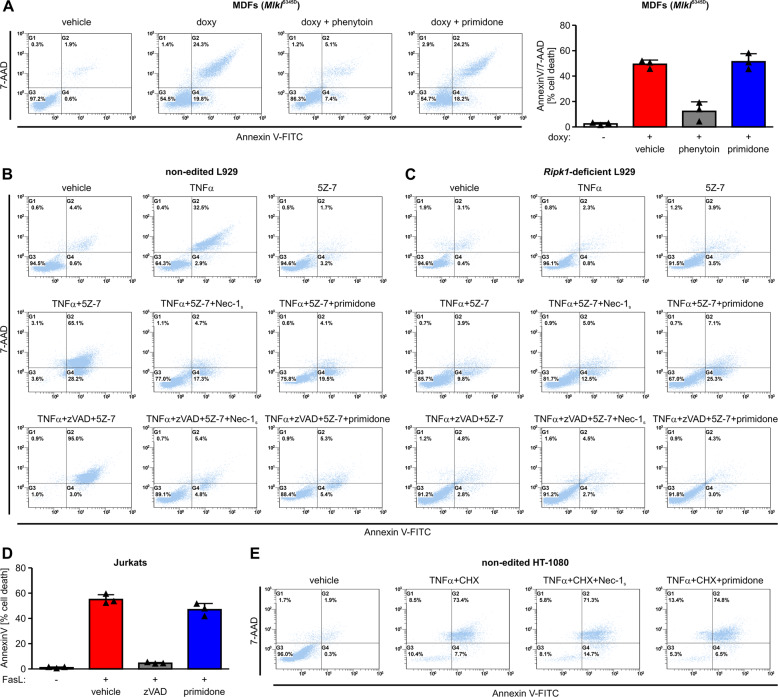
The protective effect of primidone against regulated cell death is RIPK1-mediated. **A** MDFs carrying the inducible active *Mlkl* mutant S345D (*Mlkl*^S345D^) were induced for 7 h at 37 °C with 0.5 μg/ml doxycycline (doxy) in the presence of 1 mM phenytoin or 1 mM primidone, respectively, 30 min before the addition of doxycycline. Doxycycline-induced cell death was quantified by FACS analysis using 7-AAD and phosphatidylserine accessibility (Annexin V staining) as markers. FACS dot plots of one representative experiment are shown; the adjacent graph presents the mean ± SD of three independent experiments. **B** Non-edited (parental) L929 cells were treated for 24 h at 37 °C with DMSO (vehicle), 10 ng/ml TNFα, 1 µM 5Z-7 or a combination of TNFα + 5Z-7 in the absence or presence of 50 µM Nec-1_s_ and 1 mM primidone. The combination of TNFα + 5Z-7 induced a RIPK1-dependent apoptosis (RDA), which is driven by caspase-8 inhibition (+25 µM zVAD) to RIPK1-dependent necroptotic cell death. Both cell death modalities, i.e., RDA as well as RIPK1-dependent necroptosis, are blocked by primidone. One representative approach of three independent experiments is shown. **C** Identical approaches using CRISPR/Cas9-edited *Ripk1*-deficient L929 cells confirm RDA as a promoted cell death pathway in response to TNFα + 5Z-7 stimulation. Cell death was quantified by FACS analysis using 7-AAD and phosphatidylserine accessibility (Annexin V staining) as markers. Depicted is one of three independent experiments. Fig. S1C illustrates a histogram showing the mean ± SD of the three independent experiments shown in **B**, **C**. **D** Human Jurkat cells were stimulated for 4 h at 37 °C with 100 ng/ml Fas ligand (FasL) in the absence (vehicle) or presence of 1 mM primidone. RIPK1-independent apoptotic cell death in this setting was confirmed by the addition of the pan-caspase inhibitor zVAD. The graph shows the mean ± SD of three independent experiments. **E** Human HT-1080 cells were stimulated at 37 °C for 24 h with 100 ng/ml TNFα + 1 μg/ml cycloheximide (CHX) in the absence or presence of 50 µm Nec-1_s_ and 1 mM primidone, respectively. The combination of TNFα and CHX promoted TRADD-mediated RIPK1-independent apoptosis, which was not inhibited by the addition of the RIPK1-specific inhibitor Nec-1_s_ or by primidone. Depicted is one of three independent experiments.

However, using a cell line carrying the phosphomimetic mutant *Mlkl*^S345D^ under the control of a doxycycline-inducible promoter that causes necroptosis independently of upstream signals [33], we were able to show that primidone is unable to inhibit cell death in this setting (Fig. 2A). To rule out the possibility that the mere presence of primidone induces the observed cell death independently of MLKL expression, we analyzed in a corresponding western blot to this FACS analysis the doxycycline-induced expression levels of MLKL. This approach unequivocally demonstrated that the ability of phenytoin, or rather the inability of primidone, to inhibit cell death in this RIPK1-independent setting is not due to dissimilar protein expression of MLKL in the S345D mutant (Fig. S1B). These findings prove that the mode of action of primidone differs from that of phenytoin, which influences the lipid bilayer that affects the ability of MLKL to form membrane apertures [32]. Stimulation of cells with TNFα can promote distinct cell death pathways, including RIPK1-independent apoptosis, necroptosis, and RDA [37]. As the protective effect of primidone in TNFα-induced cell death was as potent as that of the laboratory-established, but not FDA-approved, RIPK1 kinase inhibitor Nec-1_s_, we next evaluated whether the inhibitory effect of primidone in ongoing cell death requires the involvement of RIPK1. Combined treatment of L929 cells with TNFα and the TAK1 inhibitor 5Z-7-oxozeaenol (5Z-7) induced RDA, which switches by caspase-8 inhibition (addition of the pan-caspase inhibitor zVAD) to RIPK1-dependent necroptosis. Both RIPK1-dependent forms of regulated cell death (RDA and necroptosis) were blocked just as efficiently by primidone and the specific RIPK1 kinase inhibitor Nec-1_s_ (Fig. 2B and Fig. S1C). An identical approach using CRISPR/Cas9-edited *Ripk1*-deficient L929 cells in this setting confirmed that the effect of primidone in the context of regulated cell death inhibition was RIPK1-dependent (Fig. 2C and Fig. S1C). Accordingly, primidone could not block Fas ligand-induced, RIPK1-independent, apoptosis (Fig. 2D) or TRADD-mediated RIPK1-independent cell death, which is induced by TNFα/cycloheximide (CHX) (Fig. 2E). Again, CRISPR/Cas9-edited *RIPK1*-deficient HT-1080 cells in this setting confirmed that the effect of primidone in the context of regulated cell death inhibition was RIPK1-dependent (Fig. S1D).

### Primidone intervenes in complex II formation

**Fig. 3:**
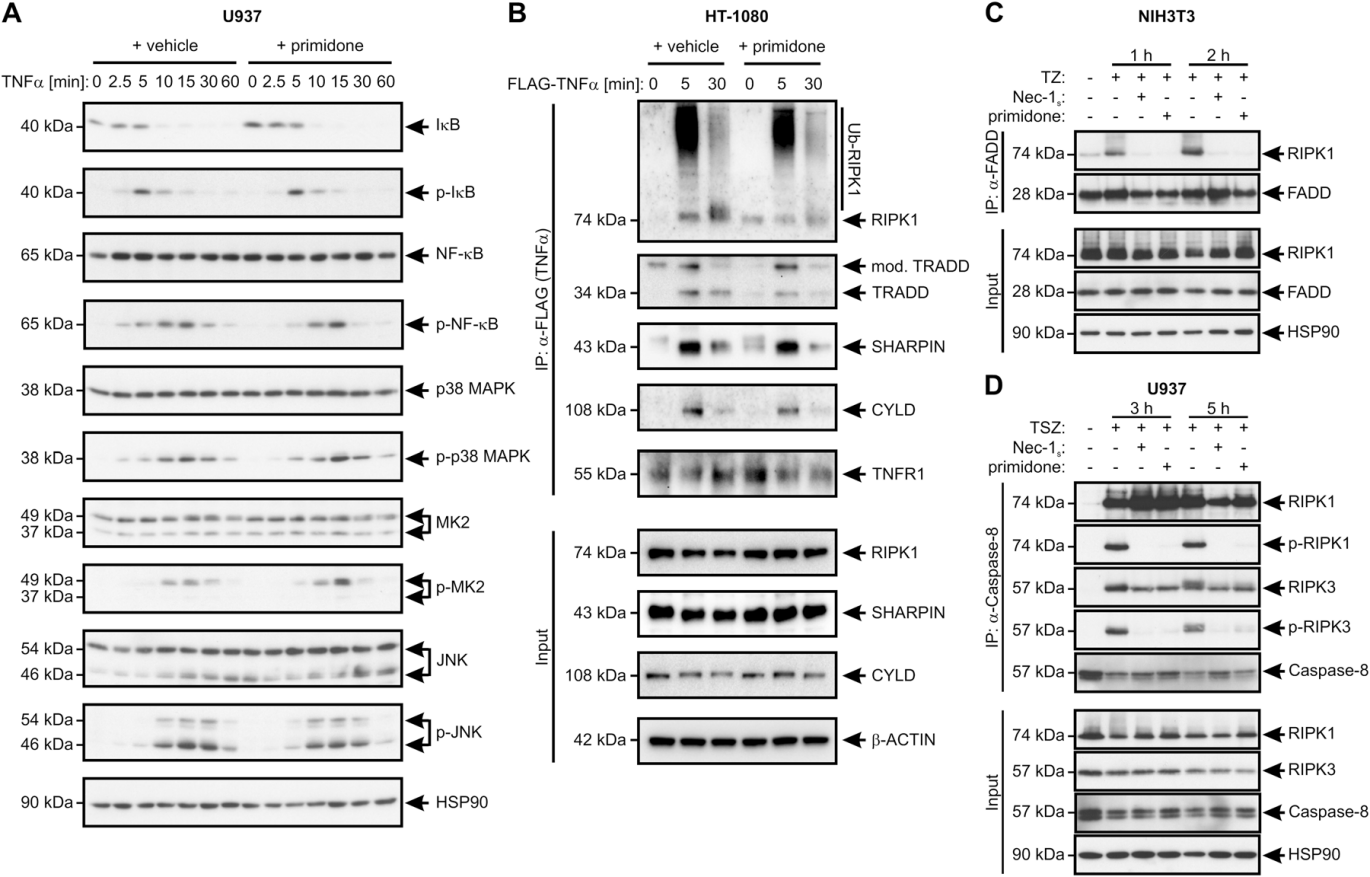
Primidone does not intervene in canonical NF-κB signaling after TNFR1 activation. **A** Human U937 cells were stimulated with 100 ng/ml TNFα in the absence or presence of 1 mM primidone for the indicated durations. Primidone was added 30 min before the addition of TNFα. Western blotting analysis of the cell lysates was performed using the indicated antibodies. **B** TNFα-induced complex I immunoprecipitation (IP) of human HT-1080 cells treated in the absence or presence of 1 mM primidone with 1 μg/ml FLAG-tagged TNFα (FLAG-TNFα) for the indicated durations, followed by anti-FLAG IP and western blotting analysis using the indicated antibodies. The results presented in **A**, **B** indicate that the biological effect of primidone is manifested in death-signaling complex II. **C** In murine cells such as NIH3T3 cells, primidone prevented the assembly of complex II. The macromolecular association of FADD, RIPK1, and RIPK3, which served as a platform for subsequent cell death was directly affected by primidone, blocking the activation (phosphorylation) of RIPK1. NIH3T3 cells were treated with 100 ng/ml TNFα + 25 μM zVAD (TZ) in the absence or presence of the RIPK1 inhibitor Nec-1_s_ and 1 mM primidone, respectively, for the indicated durations. Nec-1_s_ and primidone were added each 30 min before the addition of TNFα. TZ-induced complex II was immunoprecipitated with α-FADD antibody from cell lysates. Lysates pre-IP (Input) were also analyzed by western blotting using the indicated antibodies. **D** In human cells such as U937 cells, primidone did not prevent the assembly of the cytosolic death-inducing signaling complex II, but affected RIPK1 and RIPK3 activation, preventing TNFα-induced cell death. U937 cells were treated with 100 ng/ml TNFα + 1 μM SMAC mimetic SM-164 + 25 μM zVAD (TSZ) in the absence or presence of the RIPK1 inhibitor Nec-1_s_ and 1 mM primidone, respectively. Nec-1_s_ and primidone were each added 30 min before the addition of TNFα. The TSZ-induced complex II was immunoprecipitated with anti-caspase-8 antibody from the cell lysates. Lysates pre-IP (Input) were also analyzed by western blotting using the indicated antibodies. **A**–**D** Each blot was stripped and re-probed with an antibody against HSP90 or β-Actin (β-ACTIN) as the loading control. For each, one representative cropped blot of three independent experiments is shown.

As primidone suppressed S166 autophosphorylation in RIPK1 in response to TNFα, we hypothesized that the drug suppresses early activation of RIPK1 in the TNFR signaling complex I. Therefore, we evaluated whether primidone affects the canonical TNFα-induced NF-κB pathway. Treatment of U937 cells with TNFα in the presence and absence of primidone demonstrated that the early activation profiles (sequential phosphorylation) of IκBα, p65/RelA (NF-κB), p38 MAPK, MAPK-activated protein kinase-2 (MK2), and JNK after TNFα stimulation were unaltered (Fig. 3A). In addition, to rule out the possibility that primidone influences the recruitment of RIPK1 to TNFR1, the components of complex I underwent IP after stimulation with FLAG-tagged TNFα. As shown in Fig. 3B, the recruitment and ubiquitylation of RIPK1, and the subsequent, time-dependent recruitment of TRADD, SHARPIN, and CYLD to the TNFR1 complex upon the addition of TNFα was unchanged in the presence of primidone. In summary, these data indicate that the recruitment and chronological formation of complex I are not affected by primidone, in turn allowing a coordinated inflammatory response. The unchanged signaling of complex I suggests a role for primidone in regulating RIPK1 and complex II formation. To test this hypothesis, we analyzed the effect of primidone on RIPK1 binding to FADD, a critical step in the formation of complex II. For this purpose, we stimulated NIH3T3 cells for up to 2 h with TNFα/zVAD (TZ). In this setting, the pan-caspase inhibitor zVAD prevented RIPK1 cleavage by caspase-8 and allowed assessment of the interaction between RIPK1 and FADD in the absence or presence of primidone. Through this approach, we were able to demonstrate that primidone strongly inhibits the interaction between RIPK1 and FADD and prevents the initial formation of complex II from the outset (Fig. 3C). In human cells, such as the U937 cells we investigated, primidone behaved similarly, but complex II was not blocked during the initial assembly. Instead, the individual recruited members of the complex were blocked in the course of their activation. The phosphorylation of RIPK1, and subsequently of RIPK3, induced by TNFα/SMAC mimetic/zVAD (TSZ), was not detectable in the presence of primidone during the entire stimulation period of up to 5 h (Fig. 3D). Remarkably, in RIPK1-independent apoptosis induced by TNFα/CHX, RIPK1 was not incorporated into the FADD-associated complex II [38], which offers an explanation why primidone does not affect this type of cell death (Fig. 2E and Fig. S1D).

### Primidone is an effective inhibitor of RIPK1

**Fig. 4:**
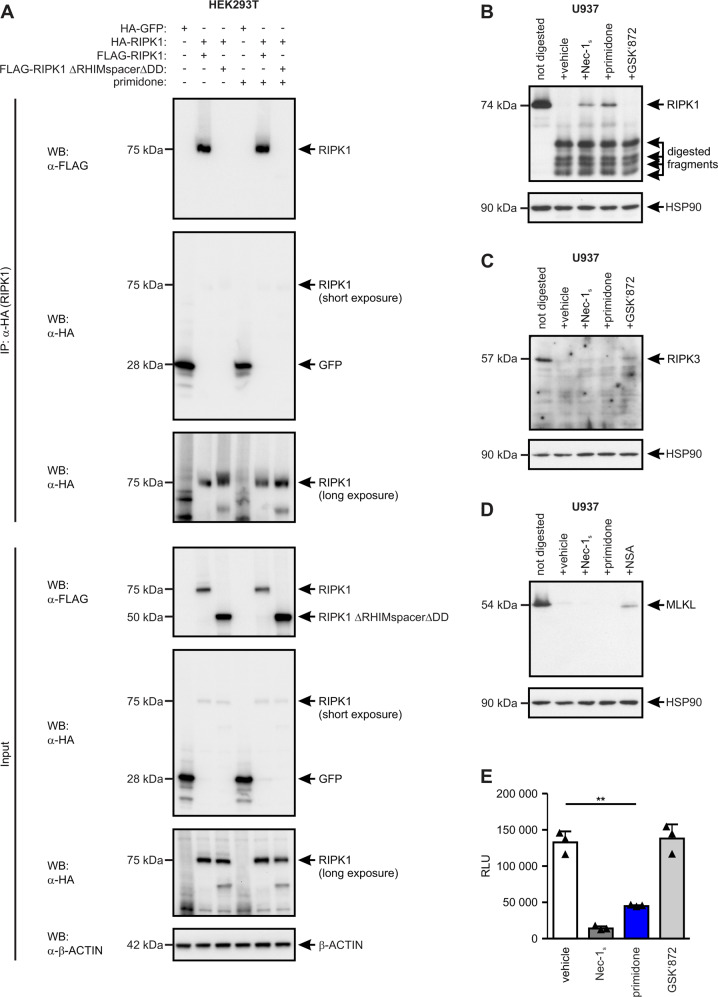
Primidone operates as a kinase inhibitor of RIPK1. **A** The possibility that the protective effect of primidone in RIPK1-mediated cell death is due to avoiding RHIM- and DD-mediated dimerization and/or oligomerization of RIPK1 was ruled out via expression experiments with tagged recombinant RIPK1 proteins. **B**–**D** The Drug Affinity Responsive Target Stability (DARTS) assay identified RIPK1 as a target of primidone. U937 cells were lysed, cleared, and incubated for 20 min at room temperature in the presence of the indicated molecules. Subsequently, protein lysates were digested for 5 min using thermolysin. **B** Increased resistance to proteolysis of RIPK1 was detected upon binding of primidone as well as Nec-1_s_ by western blotting using anti-RIPK1 antibody. Nonselective interference of small-molecules with this assay was excluded by the addition of GSK'872, a selective inhibitor of the structurally related RIPK3 that does not affect digestion of RIPK1. Simultaneous proteolysis of RIPK3 and MLKL in this setting by primidone or Nec-1_s_ was excluded by western blots using anti-RIPK3 (**C**) and anti-MLKL (**D**) antibody, respectively, in which the suitability of the DARTS assay was confirmed by the addition of GSK’872 (**C**) and NSA (**D**) as selective inhibitors of RIPK3 and human MLKL, respectively [40, 56]. All blots were stripped and re-probed with an antibody against HSP90 as the loading control. One representative cropped blot of three independent experiments each is shown. **E** The ADP-Glo^™^ Kinase Assay used to measure the kinase activity of recombinant human RIPK1 (aa 1-327) revealed that primidone is an effective kinase inhibitor of RIPK1 (RLU = relative light units). In parallel, Nec-1_s_ (a confirmed kinase inhibitor of RIPK1) and GSK’872 (a specific kinase inhibitor of RIPK3) were integrated in the assay as internal controls. The graph shows the mean ± SD of three independent experiments.

Our findings show that primidone is as effective as the well-established RIPK1 inhibitor Nec-1_s_ [39] in preventing RIPK1-dependent cell death and suggests an identical mode of action in these two molecules. To address this presumption and to rule out the possibility that primidone affects the intermolecular dimerization or oligomerization of RIPK1, which is mediated via the RHIM domain and DD of RIPK1 and believed to be essential for initiating necrosome formation [34], we used tagged RIPK1 constructs [full-length and deficient for RHIM and DD (ΔRHIMspacerΔDD)] in recombinant expression studies (Fig. 4A). The IP assays showed that primidone did not affect the interdimeric interactions facilitated by RHIM and DD. To validate the premise that RIPK1 is the specific molecular target of primidone, we subsequently performed a DARTS assay [35]. The DARTS western blotting data (Fig. 4B) clearly demonstrated that the thermolysin-mediated proteolytic digestion of RIPK1 in the presence of primidone was strongly reduced, indicating binding of the drug to RIPK1 under native conditions. In this setting, the affinity of primidone to RIPK1 was neither qualitatively nor quantitatively distinguishable from that of the selective RIPK1 inhibitor Nec-1_s_. The affinity of primidone to RIPK1 in this setting was proven by comparison with GSK'872, a selective inhibitor of the structurally related RIPK3 [40]. This specific inhibitor of RIPK3 does not bind to RIPK1 even under native conditions within a complex protein mixture. Furthermore, the appropriate DARTS assay clearly excluded a conceivable simultaneous binding of primidone to RIPK3 and MLKL, respectively, as recently described for comparable substances [41] (Fig. 4C, D).

To demonstrate conclusively that primidone interferes with the kinase activity of RIPK1, we applied the catalytically active kinase domain (aa 1-327) of a recombinant expressed and purified human GST-RIPK1 in a commercial ADP-Glo^™^ kinase assay. The luminescence-based kinase assay showed that the intrinsic RIPK1 kinase activity during the reaction phase was efficiently inhibited in the presence of primidone (Fig. 4E). A nonspecific effect of small-molecules on the assay was excluded by the addition of the RIPK3 inhibitor GSK’872, which could not block RIPK1 activity under the tested conditions. Collectively, these data emphasize that the FDA-approved drug primidone targets the kinase activity of RIPK1, which could be beneficially utilized in pathophysiological RIPK1-mediated cell death scenarios.

### Preclinical animal models confirm the efficacy of primidone

**Fig. 5:**
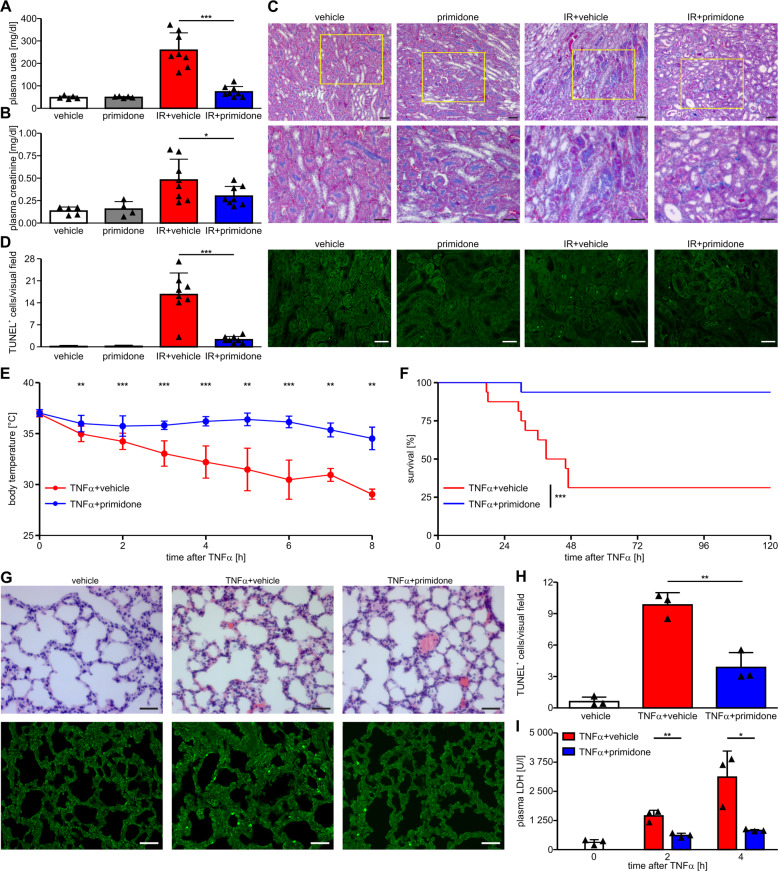
Primidone provides significant protection in different murine in vivo models. **A**, **B** In a model of renal IR, all mice (*n* = 8 per group) underwent 37 min of bilateral renal pedicle clamping, followed by 48 h of reperfusion. In the active drug group, primidone was added at a final concentration of 2.875 mM to the drinking water (renewed daily) 5 days before the onset of ischemia and until the end of the reperfusion phase. In this setting, we observed that the vehicle-treated mice in the IR group had significantly higher plasma levels of urea (**A**) and creatinine (**B**) than the primidone-treated animals. **C** Shown are representative regions of interest of the renal outer medulla stained with Masson trichrome obtained from the kidney sections of mice treated as indicated. Treatment with primidone conserved epithelial integrity, and tubular necrosis was observed only occasionally upon IRI (scale bar = 50 µm). Figure S2 shows higher-resolution images with indicators pointing to cellular debris and tubular necrosis. **D** To illustrate the protective effect of primidone at cellular level, TUNEL fluorescence assay was performed to detect and quantify cells undergoing regulated cell death (in green) in this scenario. TUNEL labeling was quantified by counting the number of positively stained cells per nonoverlapping visual field of the outer renal medulla (scale bar = 50 µm). For the SIRS investigation, all mice received a single bolus of 1 mg murine TNFα/kg body weight in a total volume of 200 µl PBS via the tail vein. In our setting, 15 min before TNFα application, the mice received a single intraperitoneal (i.p.) injection (total volume per mouse, 200 μl) of either 2.5% DMSO in PBS (vehicle) or 6.25 mg primidone/kg body weight (as indicated). **E** Mouse body temperatures (*n* = 12 per group) at distinct time points after TNFα injection. The results are the means ± SD of each group of mice. **F** Kaplan–Meier survival plot of TNFα-injected mice (*n* = 16 per group) monitored over 5 days. **G**, **H** Representative images of hematoxylin and eosin-stained lung histology sections 4 h after TNFα injection (scale bar = 50 µm). Additionally, to visualize (in green) the protective effect of primidone, we performed TUNEL fluorescence assay to detect (**G**) and quantify (**H**) cells undergoing regulated cell death. Representative images of TUNEL labeling from the lungs of mice treated for 4 h with TNFα in the absence (vehicle) or presence of primidone (*n* = 3 mice per group) are shown. TUNEL labeling was quantified by counting the number of positively stained cells per nonoverlapping visual field (scale bar = 50 µm). **I** The protective effect of primidone in this setting was corroborated by reduced plasma LDH activity in the respective mice during the initial phase (up to 4 h) of this severe disease progression.

As RIPK1 has emerged as a promising target in a wide range of human inflammatory diseases, we tested whether primidone could be used to suppress pathophysiological RIPK1-mediated cell death in renal IRI. The latter is a clinically highly relevant model because IRI is an unavoidable consequence after kidney transplantation and contributes to acute kidney injuries in various contexts [26]. In the mouse model of IRI, animals underwent 37 min of bilateral renal pedicle clamping, followed by 48 h of reperfusion. Primidone or vehicle were added to the drinking water of the mice starting 5 days before the onset of ischemia until the end of the reperfusion phase. Markers for the loss of kidney function (elevated plasma concentrations of urea and creatinine) were significantly reduced 48 h after reperfusion in primidone-treated animals (Fig. 5A, B). This finding indicates the effectiveness and therapeutic potential of primidone for treating complex diseases driven by RIPK1. A clear protective effect of primidone in this setting was also seen in Masson trichrome-stained histomicrographs of the renal outer medulla that showed better preservation of tissue integrity in the primidone-treated animals (Fig. 5C). To visualize the differences between the untreated and primidone-treated animals in this model more prominently, we included strongly magnified images of these histological sections (Fig. S2). Therein, cellular debris and tubular necrosis of single cells are additionally marked in this extension. The corresponding TUNEL fluorescence assay showed that a significantly reduced number of cells underwent regulated cell death in the primidone-treated cohort (Fig. 5D).

To substantiate the significance of primidone as an effective inhibitor of inflammatory RIPK1-dependent diseases, we extended our in vivo studies to a mouse model of SIRS, an entity of outstanding clinical relevance resembling septic shock. In experimental settings, SIRS is triggered by an intravenous injection of high-dose TNFα [42]. Our results demonstrate that pharmacological inhibition of RIPK1 by primidone significantly improves TNFα-induced hypothermia (Fig. 5E) and mortality (Fig. 5F), suggesting the potential therapeutic use of primidone in patients with hyperinflammatory disease. Hematoxylin and eosin-stained histology sections of lungs harvested 4 h after TNFα injection (Fig. 5G), and the quantification of cells that underwent regulated cell death (Fig. 5H) in this inflammatory process, visualize the protective effect of primidone. The remarkable protective effect of treatment with primidone in mice was additionally supported by significantly reduced LDH activity in the animals during the initial phase of this severe disease progression (Fig. 5I).

**Fig. 6:**
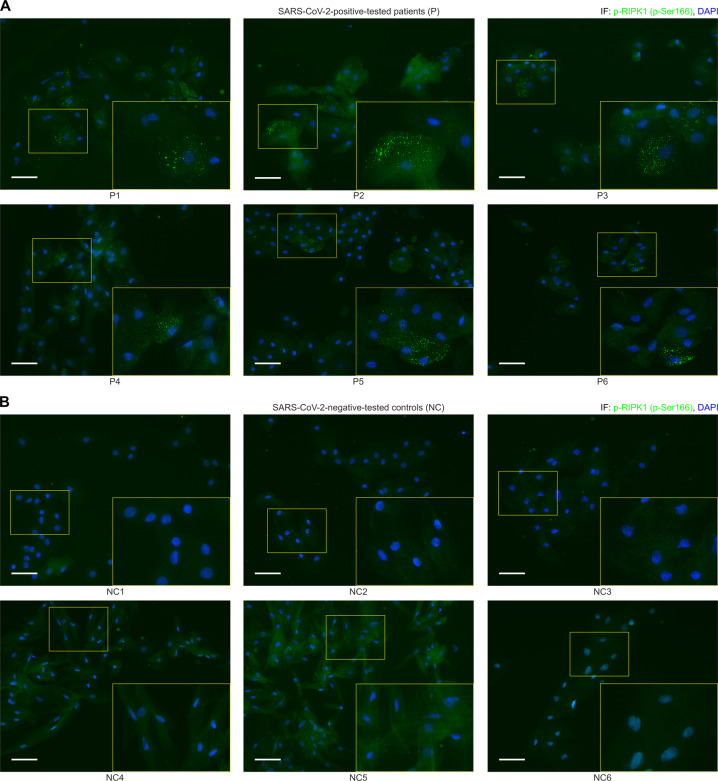
Immunohistochemical detection of p-RIPK1 in the pharyngeal epithelial cells of COVID-19 patients. **A**, **B** Overlay images of fluorescence microscopy of respiratory tract epithelial cells collected from the throat smears of symptomatic patients (P1–P6) who tested positive for SARS-CoV-2 by PCR vs. healthy negative-tested controls (NC1–NC6) stained for phosphorylated RIPK1 (in green; Fig. S3 shows the validation of p-RIPK1 antibody in ICD of human cells). DAPI (blue) was used for nuclear counterstaining. Representative staining of six tested people per group are shown (scale bar = 50 µM). The zoomed-in sections of the figure provide better visualization of the RIPK1 positivity of the affected epithelial cells. The Supplementary Information shows the case histories of the tested individuals.

During TNFα-induced SIRS, exaggerated production and secretion of pro-inflammatory cytokines and chemokines into the circulation results in the activation and increased permeability of the endothelium [23]. Interestingly, recent findings indicate that SARS-CoV-2 also induces such a hyperinflammatory response with associated cytokine release [43, 44]. In this context, after validating the suitability of a commercially available antibody (Fig. S3), we tested whether SARS-CoV-2 infection triggers RIPK1 activation in respiratory epithelial cells collected from the throat smears of symptomatic patients who had tested positive for SARS-CoV-2 by PCR. Strikingly, immunohistochemical analyses of all epithelial cell samples from the COVID-19 patients were positive for active phosphorylated RIPK1 (Fig. 6A). In complete contrast, no phospho-RIPK1-positive cells were apparent in the control samples from healthy people (Fig. 6B). Furthermore, to visualize the activation (phosphorylation) of RIPK1 in the scenario of a SARS-CoV-2 infection more prominently, we included the strongly magnified histological section (throat smear) of patient 1 (P1) in direct comparison to the negative-tested control 1 (NC1) as Fig. S4. Our data suggest that RIPK1 activation, and by implication, RIPK1-mediated ICD may contribute to the course of SARS-CoV-2 infection and could be a potential novel therapeutic target.

## Discussion

The generation of RIPK1 kinase-inactive mice enabled the investigation of the role of RIPK1 kinase-mediated regulated cell death in mouse models of inflammatory diseases [45, 46]. Concordantly, all of these reports revealed that RIPK1 is a major regulator of signaling pathways that lead to inflammation and regulated cell death, which is why RIPK1 has gained considerable interest as a drug target for treating a spectrum of human diseases, including amyotrophic lateral sclerosis (ALS), multiple sclerosis, Alzheimer’s disease, Parkinson’s disease, IBD, sepsis, viral infections, hepatitis, myocardial infarction, stroke, and IRI (reviewed in ref. [47] and our own data). Accordingly, some RIPK1 kinase inhibitors are now in phase I clinical trials for ALS and phase II clinical trials for psoriasis, rheumatoid arthritis, and ulcerative colitis [48], but to date no pharmacological inhibitor of RIPK1-mediated cell death is approved for regular clinical use. The latter was the impetus for our study, namely, evaluating FDA-approved drugs independently of their current clinical applications and assessing their utility in terms of regulated cell death inhibition. Our interest in this field stems from a much broader effort to develop a potent therapy of the cytokine release syndrome (CRS) that originates from excessive and repetitive episodes of RIPK1-driven ICD, commonly referred to as necroinflammation [49, 50].

The novel finding in our study is the identification of the potent therapeutic value of primidone, an FDA-approved drug with a 70-year safety track record, in the treatment of the aforementioned pathologies. By using several human and murine cell lines, as well as primary cells, we were able to show that primidone is an effective inhibitor of regulated cell death, preventing RDA and necroptosis. We found that the protective effect from TNFα-induced cell death was as potent as that of the well-established but not clinically applicable RIPK1 inhibitor Nec-1_s_. By using a DARTS assay, we were able to demonstrate the distinctive binding of primidone to RIPK1, which was substantiated by the ADP-Glo^™^ Kinase Assay showing that the kinase activity of RIPK1 was inhibited specifically by primidone. This selectivity of primidone is certainly different from comparably potent substances such as the recently described “compound 2”, which can bind and inhibit at different levels all three necrosome members (RIPK1, RIPK3, MLKL) simultaneously [41]. The dynamic assembly of the membrane-bound signaling complex I, which is rapidly induced upon TNFα binding to its corresponding receptor and the subsequent activation of the NF-κB pathway, was not impaired by primidone. Furthermore, in the course of our studies, we successfully used primidone in vivo to suppress RIPK1-driven cell death in renal IRI.

The kinase activity of RIPK1 mediates hypothermia and mortality in a mouse model of TNFα-induced shock, reflecting the hyperinflammatory state of SIRS [51]. By using the aforementioned TNFα-induced shock model, we have proven that RIPK1 activation and RIPK1-mediated cell death, which plays an important role in inflammatory syndromes, can be efficiently prevented by primidone. As primidone is FDA-approved, it could therefore be a promising drug for targeting similar diseases in humans.

Recent findings have highlighted necroptosis as the predominant form of ICD in viral infections, such as in the case of the respiratory influenza A and neuropathogenic coronaviruses [52]. Moreover, a current study by Simpson et al. proves for the first time that respiratory syncytial virus infection induces the release of the inflammatory nuclear alarmin HMGB1 (high mobility group box 1). In this scenario, this is the proximate consequence of necroptotic dying airway epithelial cells, and the prevention of ICD, more precisely the inhibition of the activated necrosome members RIPK1 and MLKL, ameliorates viral bronchiolitis-associated pathologies, at least in mice, preventing the later progression to asthma [53]. Furthermore, the original SARS coronavirus, termed SARS-CoV-1, promotes multiple forms of necrotic cell death, such as RIPK3-dependent necroptosis [54]. We can now show that SARS-CoV-2 infection triggers RIPK1 activation in respiratory tract epithelial cells isolated from the throat smears of symptomatic patients who had tested positive for SARS-CoV-2 by PCR. Immunohistochemical analyses of pharyngeal epithelial cell samples from COVID-19 patients revealed high rates of cells positive for active phosphorylated RIPK1. In contrast, activated phospho-RIPK1 was negligible in the control samples from healthy people, suggesting that RIPK1 activation, and by implication, RIPK1-induced ICD, contributes to the course of SARS-CoV-2 infection.

Consistent with its role in inflammation and disease in mouse models, our data suggest that SARS-CoV-2-induced RIPK1 activation may also contribute to COVID-19-associated CRS, which is supported by evidence that nsp12, a viral RNA polymerase for SARS-CoV-2 replication, binds RIPK1 in the COVID-19 interactome [55]. The finding that RIPK1 is activated during COVID-19, together with its implication in the pathogenesis of many human inflammatory diseases, makes RIPK1 an attractive drug target for ameliorating or suppressing the lethal CRS associated with SARS-CoV-2 infection. Therefore, our data warrant a clinical trial to assess the benefit of RIPK1 inhibition in patients with COVID-19. An application to conduct a clinical trial using primidone in a subgroup of SARS-CoV-2-positive patients with acute respiratory distress syndrome is currently being submitted to the EudraCT (European Union Drug Regulatory Authorities Clinical Trials Database). As the safety, tolerability, pharmacokinetics, and pharmacodynamics of primidone are well-documented, this drug would be readily available for clinical application in diseases that are triggered through pathological RIPK1 activation. It would be logical to test additional pharmaceuticals that are approved for other indications and already available on a large scale. Of course, all predicted novel compounds still require experimental testing, but SARS-CoV-2 places drug repurposing on the fast track.

## Supplementary information

Supplementary Information

Figure S1

Figure S2

Figure S3

Figure S4
