# Supplementary material for: Primidone blocks RIPK1-driven cell death and inflammation
Source: Cell Death Differ. 2020 Dec 3;28(5):1610–26. doi: 10.1038/s41418-020-00690-y (PMC7712602; doi:10.1038/s41418-020-00690-y)
Supplement: Supplementary file 1 — Supplementary Information [file 41418_2020_690_MOESM1_ESM.docx]

**Supplementary Information**

**Primidone blocks RIPK1-driven cell death and inflammation**

**Case histories of patients**

**Case history of SARS-CoV-2-positive-tested patient 1 (P1)**

A 69-year-old man presented to our clinic after referral from his primary care physician because of fever up to 39°C and progressive malaise. Symptoms had begun with a feeling of fever and a dry cough ten days before presentation. Additionally, he reported dysgeusia and night sweats, but no shortness of breath. He tested positive for SARS-CoV-2 infection upon arrival, and a sample for this study was taken one day later. His only pre-existing medical condition was arterial hypertension. His breathing rate was 20 breaths per min, heart rate was 70 beats per min, blood pressure was 130/80 mmHg, and body temperature was 38.3°C. His blood oxygen saturation was 96% under ambient air. Physical examination revealed fine crackles at the base of both lungs but was otherwise unremarkable. Chest X-rays showed small infiltrates at the base of the left lung. Laboratory results showed lymphocytopenia and increased inflammatory markers (C-reactive protein, IL-6, ferritin, D-dimer). Symptomatic treatment with acetaminophen was initiated. During the course of treatment, his symptoms decreased gradually, but dysgeusia persisted. After two weeks’ treatment and negative testing for SARS-CoV-2, he was discharged home.

**SARS-CoV-2-positive-tested patient 2 (P2)**

A 49-year-old man was transferred to our clinic from another hospital. He reported fevers of up to 40°C, progressive cough, and nasal discharge that had persisted for four days. He tested positive for SARS-CoV-2 infection at another hospital one day before admission, and repeat testing upon admission at our hospital showed borderline-positivity. A sample for this study was obtained the next day. His pre-existing conditions were limited to diabetes mellitus type II. Upon presentation, he complained of mild shortness of breath with a breathing rate of 19 per min but appeared otherwise healthy. His blood pressure was 145/80 mmHg, heart rate was 80 beats per min, and body temperature was 38.9°C. Auscultation revealed fine crackles at the base of both lungs, but physical examination was otherwise unremarkable. Blood oxygen saturation was 93% under supplementation of 2 l/min oxygen flow through a nasal canula. Chest X-rays showed infiltrates at the base of both lungs. His laboratory results were remarkable for increased markers of inflammation (C-reactive protein, IL-6, ferritin, D-dimer) and lymphopenia. Symptomatic treatment included acetaminophen and intravenous application of crystalloid solutions. Five days after admission, the patient reported increasing shortness of breath. Arterial blood gas analysis showed hypoxemia with oxygen partial pressure of 62 mmHg under supplementation of 3 l/min oxygen flow through a nasal canula. Oxygen supplementation was switched to a non-rebreather mask and was increased to 4 l/min oxygen flow. Subsequently, the dyspnea resolved over five days, when supplementation of oxygen was ceased. The patient’s condition improved under continued supportive care. After two weeks of treatment and negative testing for SARS-CoV-2, he was discharged home.

**SARS-CoV-2-positive-tested patient 3 (P3)**

A 64-year-old man was admitted to our hospital after collapsing at a nearby campground. He regained consciousness rapidly after emergency medicine technicians diagnosed hypoglycemia (glucose, 49 mg/dl; reference range, 76-108 mg/dl), and intravenous glucose solution was applied. No other symptoms were noted, particularly no dysgeusia, cough, fever, or dyspnea. The breathing rate was 16 breaths per min, blood pressure was 142/76 mmHg, heart rate was 78 beats per min, and body temperature was 36.7°C. Physical examination was unremarkable except for moderate obesity, and laboratory results as well as radiology studies showed no signs of infection or inflammation. SARS-CoV-2 infection was detected upon routine testing when the patient was admitted to the hospital, and he remained isolated for two days. His pre-existing medical conditions were limited to diabetes mellitus type I and obesity. As no further serious symptoms developed and his blood glucose levels had stabilized, the patient was released 72 h after initial presentation into home quarantine with instructions to present to his primary care physician for a follow-up appointment.

**SARS-CoV-2-positive-tested patient 4 (P4)**

A 75-year-old man was initially evaluated in the emergency room of another hospital where he presented with a dry cough and severe difficulty breathing. His symptoms had gradually increased over the course of two days, and a fever up to 39°C had developed. He tested positive for SARS-CoV-2 infection upon arrival. His pre-existing medical conditions included diabetes mellitus type II, arterial hypertension, asthmatic lung diseases, hypothyroidism, and benign prostatic hyperplasia. Physical examination showed apparent respiratory distress with crackles over the bases of both lungs. His breathing rate was 30 breaths per min, blood pressure was 110/65 mmHg, heart rate was 96 beats per min, and temperature was 38.4°C. As his condition continued to deteriorate, he was placed on mechanical ventilation after successful intubation. A prolonged stay on the intensive care unit ensued. After six weeks of treatment, he was weaned and successfully extubated, but the severe cough returned, and a computed tomography (CT)-scan of the chest showed radiological signs of COVID-19 without any hints of superinfection. Repeat testing showed continued positivity for SARS-CoV-2, and a sample was obtained for this study. A high-sensitivity troponin test showed a newly elevated troponin-I level of 12,400 ng/l (reference range < 45 ng/l), indicating acute myocardial damage. The electrocardiogram (ECG or EKG) showed no pathological signs. Treatment with 500 mg acetylsalicylic acid (ASS) and heparin was initiated, but invasive diagnostics had not been conducted at the time of submission, and neither myocardial infarction nor myocarditis had been ruled out.

**SARS-CoV-2-positive-tested patient 5 (P5)**

A 41-year-old woman was admitted to our hospital because of joint pain, slight fevers, and cough. She had recently been visited by her sister who later tested positive for SARS-CoV-2 infection. Besides feeling very tired, she did not notice any further symptoms, particularly no dyspnea or dysgeusia. The only pre-existing medical condition was minor thalassemia. Upon admission, her breathing rate was 16 breaths per min, blood pressure was 110/70 mmHg, heart rate was 90 beats per min, and body temperature was 37.7°C. Physical examination was unremarkable except for moderate obesity. Her laboratory results showed mild microcytic anemia, modest lymphopenia, as well as moderately elevated C-reactive protein levels. Chest X-rays showed no signs of infection or inflammation. SARS-CoV-2 infection was detected when the patient was admitted to the hospital and a sample was obtained for this study. The patient remained in stable condition and was released to self-quarantine at home after two days of symptomatic treatment.

**SARS-CoV-2-positive-tested patient 6 (P6)**

A 32-year-old man was transferred to our clinic from another hospital. He had previously been on vacation in Eastern Europe and developed severe difficulty breathing, fevers up to 39.5°C, and dysgeusia shortly after his return to Germany. He had tested positive for SARS-CoV-2 infection at another hospital, where physical examination showed apparent respiratory distress with crackles over the bases of both lungs. His breathing rate was 32 breaths per min, blood pressure was 124/74 mmHg, heart rate was 98 beats per min, and temperature was 38.9°C. CT-scan of the chest showed prominent infiltrates at the base of both lungs, and laboratory markers of inflammation (C-reactive protein, IL-6, ferritin, D-dimer) were markedly increased. Due to his increasing respiratory distress, a decision was made to transfer the patient to the intensive care unit of our hospital, where repeat testing for SARS-CoV-2 was positive, and a sample was obtained for this study. His breathing rate increased to 40 breaths per min and blood oxygen saturation decreased to 90%, but intubation was not deemed necessary, and he improved under ventilation with continuous positive airway pressure and treatment with dexamethasone.

**Controls**

The controls did not show any signs of infection, in particular, no cough, dysgeusia, or sneezing, and no elevated inflammatory markers (C-reactive protein, IL-6, ferritin, D-dimer).

**SARS-CoV-2-negative-tested control 1 (NC1)**

A 43-year-old man with no pre-existing medical conditions.

**SARS-CoV-2 negative-tested control 2 (NC2)**

A 57-year-old woman with no pre-existing medical conditions.

**SARS-CoV-2-negative-tested control 3 (NC3)**

A 41-year-old man with no pre-existing medical conditions.

**SARS-CoV-2-negative-tested control 4 (NC4)**

A 79-year-old woman with no pre-existing medical conditions.

**SARS-CoV-2-negative-tested control 5 (NC5)**

A 29-year-old man with no pre-existing medical conditions.

**SARS-CoV-2-negative-tested control 6 (NC6)**

A 58-year-old man with no pre-existing medical conditions.

**Supplementary figure legends**

**Supplementary Fig. S1 The protective effect of primidone against regulated cell death is RIPK1-mediated.** (A) Chemical structure of primidone (C_12_H_14_N_2_O_2_). (B) Doxycycline-induced expression of MLKL in mutated mouse dermal fibroblasts (MDFs) was not affected by primidone. MDFs carrying the inducible active *Mlkl* mutant S345D (*Mlkl*^S345D^) were induced for 7 h at 37°C with 0.5 μg/ml doxycycline (doxy) in the presence of 1 mM phenytoin or 1 mM primidone, respectively, 30 min before the addition of doxycycline. Identical samples to those analyzed by FACS (Fig. 2A) were used for detecting whole MLKL expression by western blotting using an anti-MLKL antibody (MABC604, clone 3H1). The blot was redeveloped with an antibody against β-Actin (β-ACTIN) as the loading control. (C) Non-edited (parental) L929 and CRISPR/Cas9-edited *Ripk1*-deficient L929 cells were treated for 24 h at 37°C with DMSO (vehicle), 10 ng/ml TNFα, 1 µM 5Z-7-oxozeaenol (5Z-7) or a combination of TNFα + 5Z-7 in the absence or presence of 50 µM Nec-1_s_ and 1 mM primidone as indicated. The combination of TNFα + 5Z-7 induced a RIPK1-dependent apoptosis (RDA) which was driven by caspase-8 inhibition (+ 25 µM zVAD) to RIPK1-dependent necroptotic cell death. RDA as well as RIPK1-dependent necroptosis were blocked by primidone. The CRISPR/Cas9-edited *Ripk1*-deficient L929 cells confirmed RDA as a promoted cell death pathway in response to TNFα + 5Z-7 stimulation and emphasized primidone as an RIPK1 kinase inhibitor. Cell death was quantified by FACS analysis using 7-amino-actinomycin D (7-AAD) and phosphatidylserine accessibility (Annexin V staining) as markers. Histogram shows the mean ± SD of the three independent experiments. (D) CRISPR/Cas9-edited *RIPK1*-deficient HT-1080 cells were treated for 24 h at 37°C with DMSO (vehicle) or 100 ng/ml TNFα + 1 μg/ml cycloheximide (CHX) in the absence or presence of 50 µm Nec-1_s_ and 1 mM primidone, respectively. The combination of TNFα and CHX promoted a TRADD-mediated, RIPK1-independent apoptosis which was not inhibited by the addition of the RIPK1-specific inhibitor Nec-1_s_ or by primidone. Cell death was quantified by FACS analysis using 7-AAD and phosphatidylserine accessibility (Annexin V staining) as markers. One representative approach of three independent experiments is depicted.

**Supplementary Fig. S2 Primidone provides protection from renal IRI.** Shown are enlargements of the images illustrated in Fig. 5C. Cellular debris (yellow arrowheads) and tubular necrosis (black arrows) resulting from ischemia-reperfusion (IR) damage are highlighted. The significant therapeutic effect of primidone in this model was apparent by the conserved epithelial integrity of cells in the appropriately treated animals (scale bar = 50 µm).

**Supplementary Fig. S3 RIPK1-dependent inflammatory cell death of human cells can be detected by immunofluorescence staining of RIPK1.** For validating the suitability of a commercially available human phospho-RIPK1 antibody in an inflammatory cell death scenario we treated human HT-29 cells for 4 h at 37°C with 100 ng/ml TNFα + 1 μM SMAC mimetic SM164 + 25 μM zVAD (TSZ) in the absence or presence of the established RIPK1 inhibitor Nec-1_s_ (50 µM) or 1 mM primidone, respectively. Nec-1_s_ and primidone were added 30 min before stimulation with TNFα. Activation of RIPK1 in the ongoing cell death process was analyzed by immunocytochemistry using a phospho-RIPK1 (Ser166) antibody (p-RIPK1 = green). DAPI (blue) was used for nuclear counterstaining. Shown are representative images from one of three independent experiments*.* The zoomed-in sections as part of the figure provide better visualization of the RIPK1 positivity of the affected necroptotic dying cells (scale bar = 50 µM).

**Supplementary Fig. S4 Immunohistochemical detection of p-RIPK1 in pharyngeal epithelial cells.** For better visualization of the RIPK1 positivity of the affected epithelial cells, shown are enlargements of two images that were illustrated in lower resolution in Fig. 6. Shown are images of fluorescence microscopy of respiratory tract epithelial cells collected from the throat smears of symptomatic patient 1 (**P1**), who tested positive for SARS-CoV-2 by PCR vs. a healthy negative-tested control (**NC1**) stained for phosphorylated RIPK1 (p-RIPK1 = green). DAPI (blue) was used for nuclear counterstaining (scale bar = 50 µM).
