# Supplementary figures and images for: Primidone blocks RIPK1-driven cell death and inflammation
Source: Cell Death Differ. 2020 Dec 3;28(5):1610–26. doi: 10.1038/s41418-020-00690-y (PMC7712602; doi:10.1038/s41418-020-00690-y)

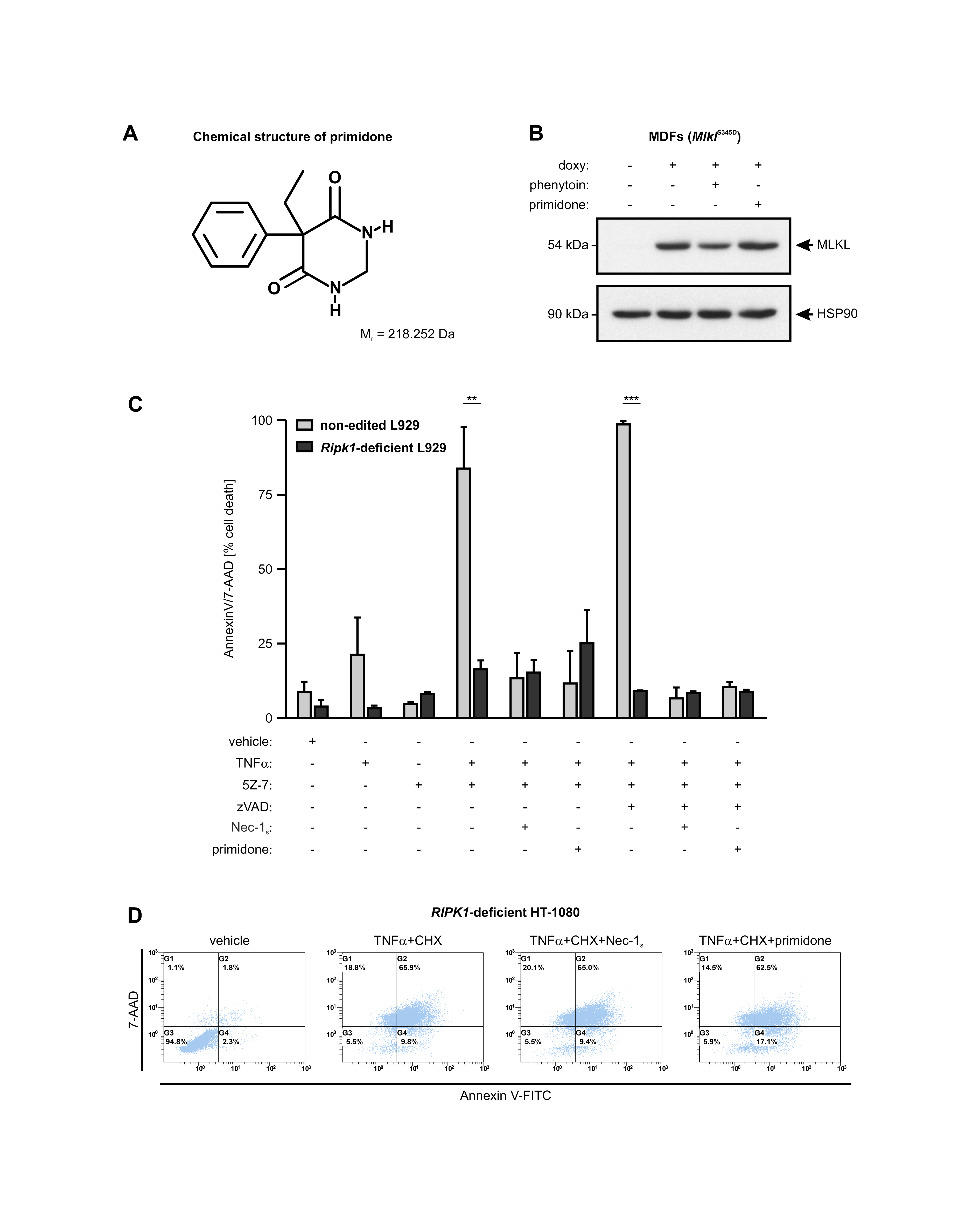

Supplement: Supplementary file 2 — Figure S1 [file 41418_2020_690_MOESM2_ESM.tif]

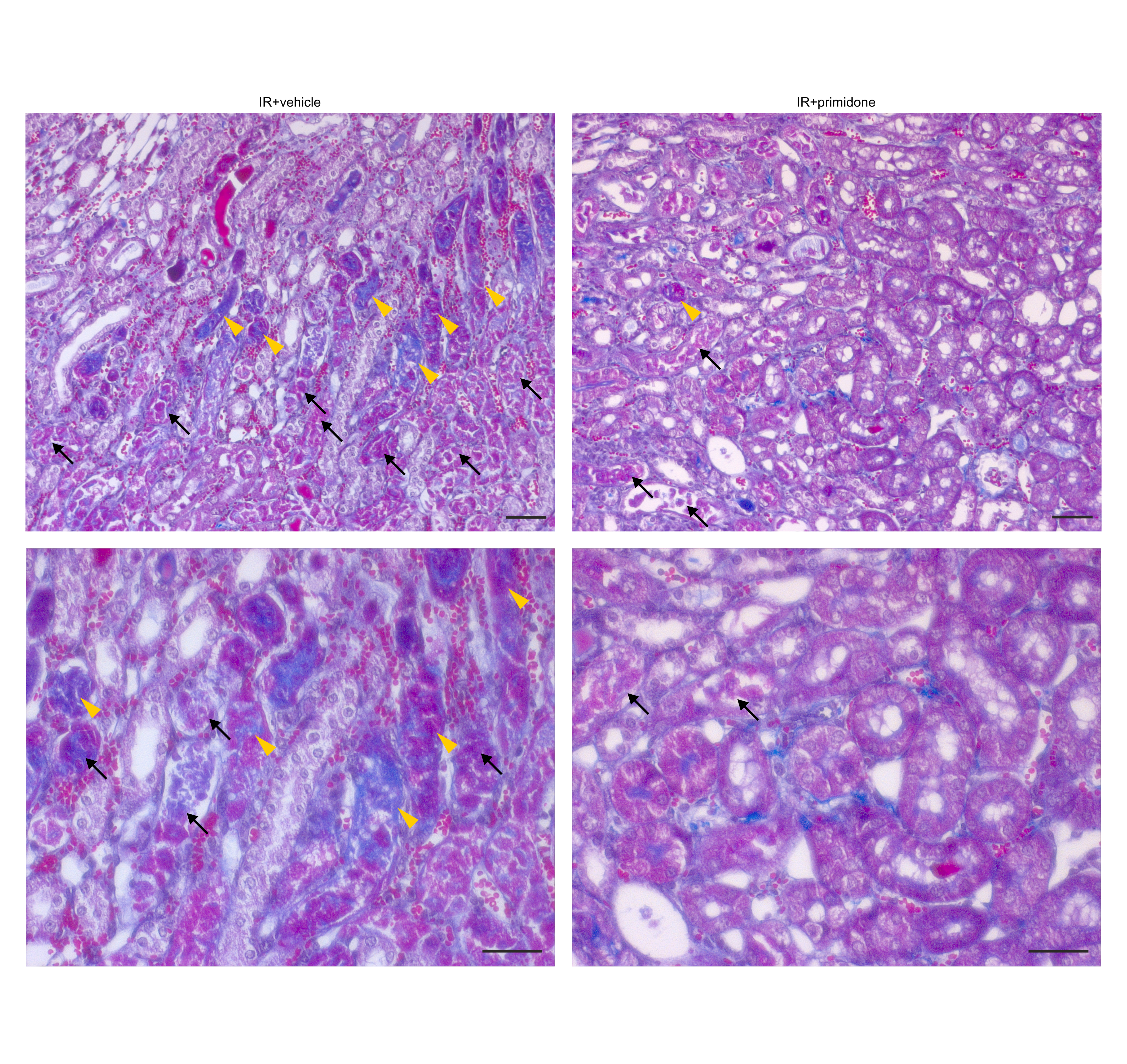

Supplement: Supplementary file 3 — Figure S2 [file 41418_2020_690_MOESM3_ESM.tif]

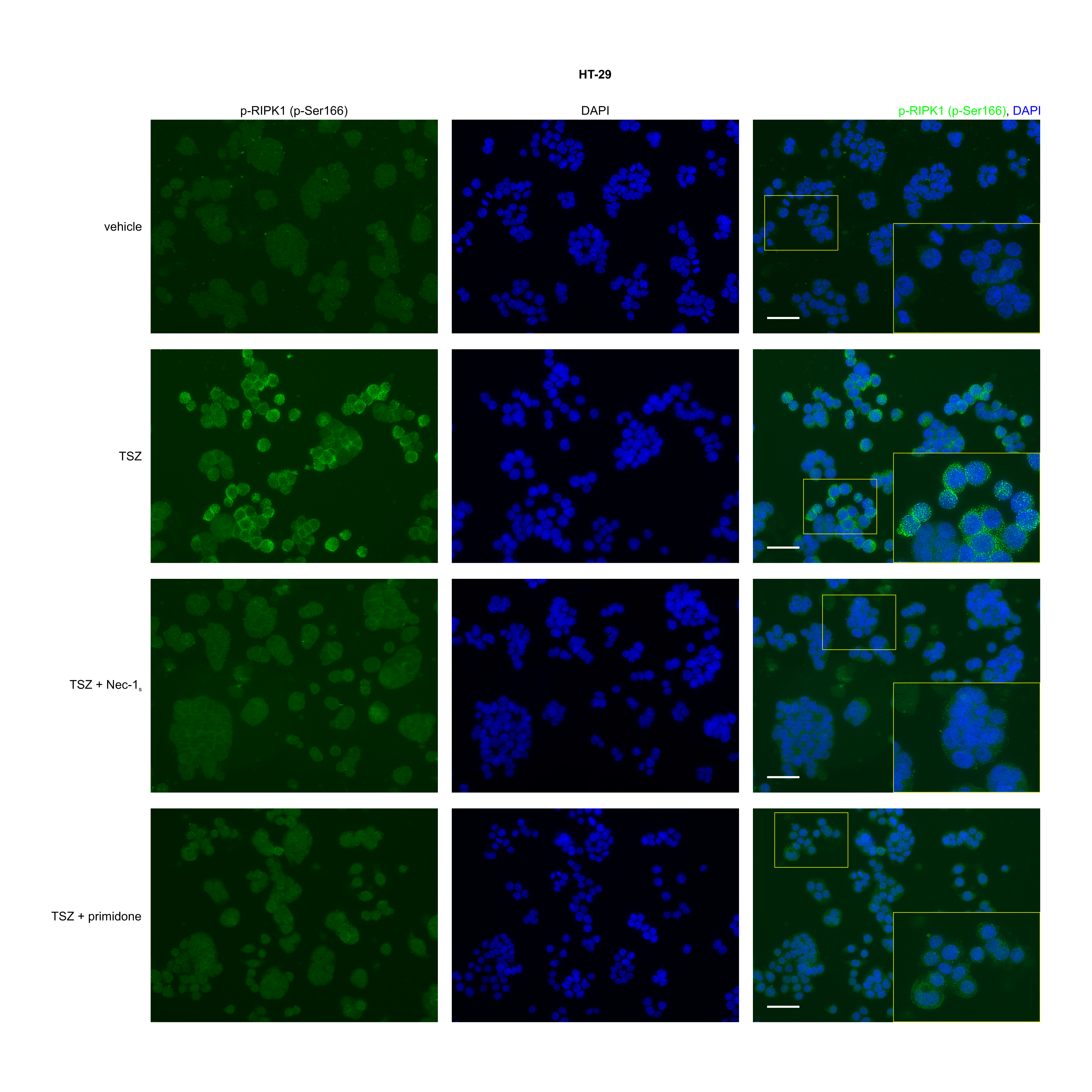

Supplement: Supplementary file 4 — Figure S3 [file 41418_2020_690_MOESM4_ESM.tif]

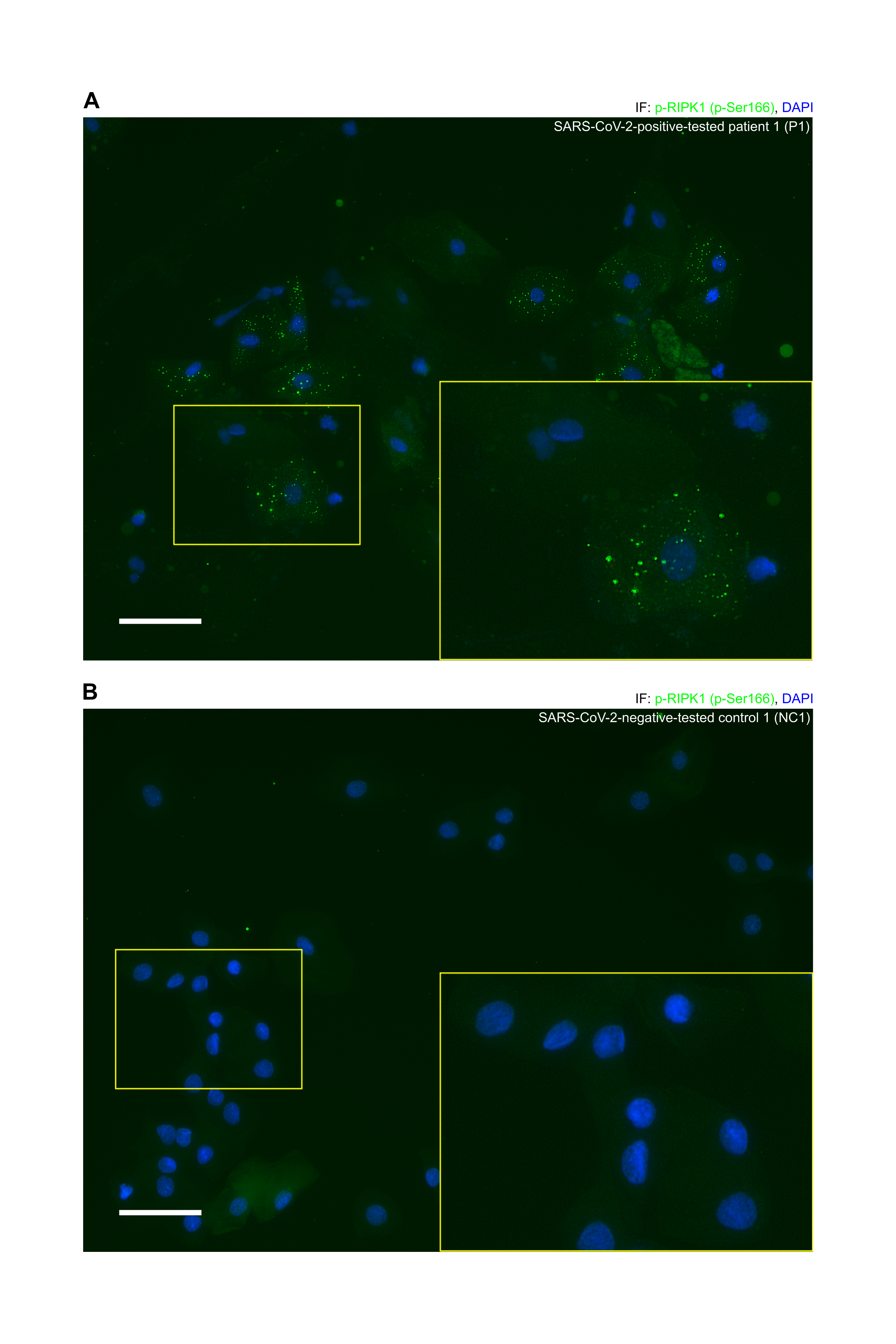

Supplement: Supplementary file 5 — Figure S4 [file 41418_2020_690_MOESM5_ESM.tif]
